# The complete spectrum of pentalogy of Cantrell in one of a set of dizygotic twins: A case report of a rare congenital anomaly

**DOI:** 10.1097/MD.0000000000025470

**Published:** 2021-04-09

**Authors:** Zlatan Zvizdic, Irmina Sefic-Pasic, Amira Mesic, Sabina Terzic, Semir Vranic

**Affiliations:** a Clinic of Pediatric Surgery; b Department of Radiology; c Department of Anesthesiology and Reanimation; d Pediatric Clinic, University Clinical Center Sarajevo, Sarajevo, Bosnia and Herzegovina; e College of Medicine; f Biomedical and Pharmaceutical Research Unit, Qatar University Health, Qatar University, Doha, Qatar.

**Keywords:** congenital anomalies, diagnosis, pentalogy of Cantrell, treatment, twins

## Abstract

**Rationale:**

Pentalogy of Cantrell (POC) is an extremely rare syndrome with an estimated incidence of 1:65,000 to 200,000 live births. Its complete form includes a midline epigastric abdominal wall defect, defects affecting the lower sternum, anterior diaphragm, diaphragmatic pericardium, and various intracardiac defects.

**Patient concerns:**

We report a case of complete POC affecting only the first-born of a set of premature dizygotic twins.

**Diagnosis:**

A giant omphalocele with an eviscerated liver and bowel on prenatal, obstetric ultrasonography at 24 gestational weeks was observed. At birth, physical examination confirmed a massive (10 × 8 cm) epigastric omphalocele in which a significant part of the liver was seen. A postnatal echocardiogram revealed the presence of an ostium secundum atrial septal defect, perimembranous ventricular septal defect, and moderate pulmonary stenosis. X-ray showed an abnormal intrathoracic positioned stomach, which was confirmed with a plain x-ray of the upper intestinal tract with hydrosoluble contrast. Computed tomography (CT) scan revealed the sternum's absence and a close connection between the pericardial sac and the stomach wall.

**Interventions:**

The patient underwent surgical intervention at 18 days of age.

**Outcomes:**

Despite adequate and appropriate postoperative treatment, the baby rapidly deteriorated and died 72 hours after surgery.

**Lessons:**

POC is a complex, high-mortality syndrome whose management requires a multidisciplinary approach and meticulous planning. Despite all efforts, POC carries a poor prognosis, particularly in patients affected by its complete form.

## 1 Introduction

Pentalogy of Cantrell (POC) is an extremely rare syndrome with an estimated incidence of 1:65,000 to 200,000 live births, affecting men and women at a ratio of 1.35:1.^[1,2]^ Some 250 cases have been reported so far in the literature, mostly in the western countries (the United States and Europe).^[1]^ POC is a constellation of five congenital defects, including a midline epigastric abdominal wall defect, defects affecting the lower sternum, anterior diaphragm, diaphragmatic pericardium, and various intracardiac defects.^[3]^ However, the hallmark of POC is an omphalocele associated with ectopia cordis (EC). In some cases, the spectrum of the POC had not been present in a complete manner. Based on that, Toyama^[4]^ described the probable syndrome variant that includes 4 defects, intracardiac and ventral abdominal wall abnormalities, and the incomplete syndrome with various combinations of defects present, but always with sternal abnormalities. POC is associated with a high mortality rate that increases in cases of complex heart defects, pulmonary hypoplasia, associated malformations, and the existence of a complete presentation of this congenital disorder.^[5,6]^

Herein, we report an extremely rare case of complete POC affecting only the first-born of a set of premature dizygotic twins.

## 2 Case report

A 33-year-old gravida 1, para 0, abortus 0, was referred to the obstetrics service at 34 gestational weeks for elective cesarean section (CS) due to the finding of a giant omphalocele with an eviscerated liver and bowel on prenatal, obstetric ultrasonography (US) at 24 gestational weeks in one of a set of dizygotic twins. The second twin was ultrasonographically normal. The parents were counseled regarding ultrasound findings. The woman was also diagnosed with preeclampsia and gestational diabetes mellitus, and methyldopa (250 mg orally twice a day) was administered. The family history was negative for congenital anomalies or genetic abnormalities. There was no history of consanguinity.

**Figure 1:**
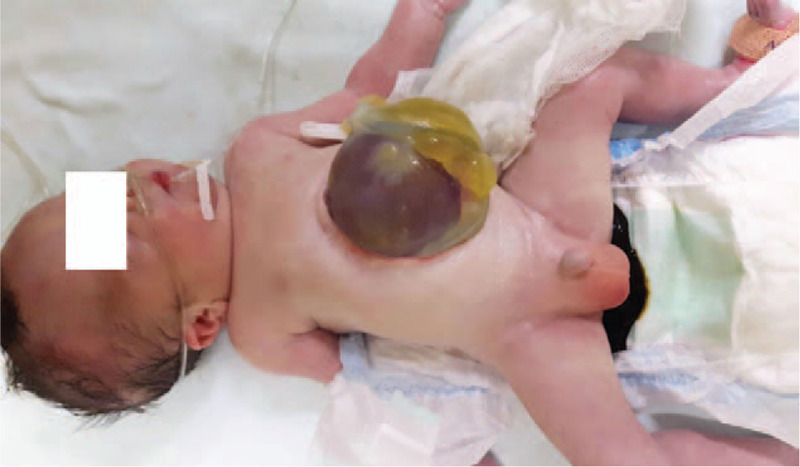
The postnatal photograph of a neonate depicts a huge cephalic, a liver-containing omphalocele.

At delivery, the affected male twin had a birth weight of 2.050 g with the Apgar score of 4/6 in the first and fifth minute, respectively. The infant was found to be cyanotic and hypotonic with low respiratory effort. His peripheral oxygen saturation was 90% via pulse oximetry, respiratory rate was 48 breaths per minute, the heart rate was 158 bpm, and blood pressure was 64/32 mm Hg. Basic blood tests were normal. Physical examination confirmed a massive (10 × 8 cm) cephalic (epigastric) omphalocele in which a significant part of the liver was observed (Fig. 1). The site of the omphalocele was covered with a saline-soaked gauze pad. Following admission to the neonatal intensive care unit (NICU), the baby was placed on nasal prong oxygen at 2 L/min. Antibiotics (ampicillin and amikacin) were also administered. The baby received daily dressing changes.

**Figure 2:**
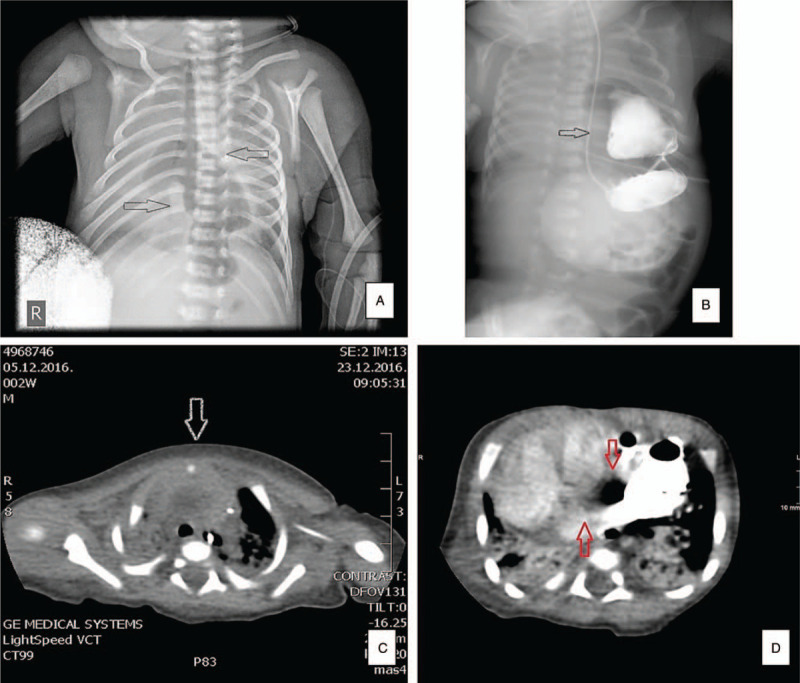
A–D: A: Plain chest X-ray with transparent, air-filled shadow positioned in the midline of the thorax-abnormal position of the stomach (white arrows); B: AP view—a plain X-ray of the upper intestinal tract with hydrosoluble contrast in abnormally intrathoracic positioned stomach (white arrow); C: Axial CT scan indicates one small ossification center of the sternum (incomplete ossification of the sternum) (white arrow); D: Coronal CT scan where is shown a close connection between pericardial sac and stomach wall (stomach filled with an oral contrast) (red arrows). CT = computed tomography.

A postnatal echocardiogram demonstrated an ostium secundum atrial septal defect (ASD), perimembranous ventricular septal defect (VSD), and moderate pulmonary stenosis. A plain chest X-ray showed an abnormal intrathoracic positioned stomach (Fig. 2A), which was confirmed with a plain X-ray of the upper intestinal tract with hydrosoluble contrast (Fig. 2B). Computerized tomography (CT) scan findings showed the absence of the sternum with the only small sternal ossification center (Fig. 2C) and a close connection between the pericardial sac and the stomach wall (Fig. 2D).

**Figure 3:**
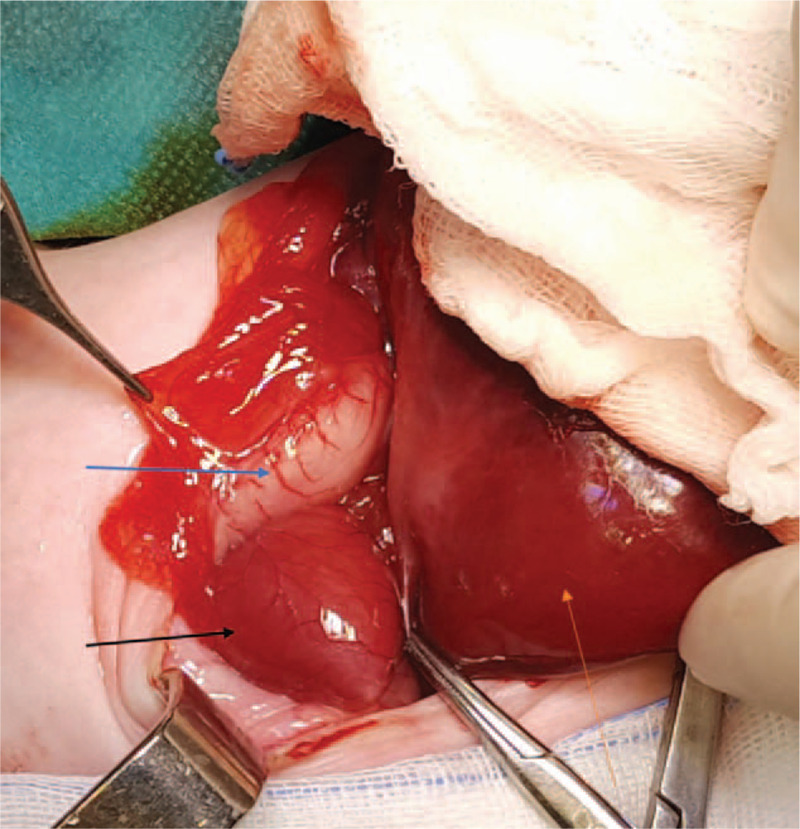
Surgical intraoperative view: the stomach (blue arrow) after reduction from anterior diaphragmatic hernia cavity, ectopic heart (black arrow), and centrally located eviscerated liver (orange arrow).

During the first 2 weeks of life, the baby had multiple bradycardia episodes and low oxygen saturation, and the surgery was postponed until the patient's relative stabilization was achieved. The patient underwent surgical intervention at 18 days of age. After dissection of the omphalocele sac from the skin edges, the ectopic heart in the right upper abdominal cavity, bilateral anterior diaphragmatic defect with herniation of the transverse colon and the stomach into the thoracic cavity, and centrally located eviscerated liver were noted (Fig. 3). After reducing the transverse colon into the abdominal cavity, defects in the diaphragmatic pericardium and the right and the left hemidiaphragm were reconstructed and sutured. Due to the abdominal wall defect (10 cm in diameter) and the liver's central position, primary fascial reconstruction was not an option. The closure of the omphalocele defect was performed using a silo bag.

After the procedures, the patient was taken intubated to the NICU. Despite adequate and appropriate ventilation and high doses of dobutamine and noradrenaline, the patient developed inadequate oxygenation, progressive hypotension, and oliguria. An adequate increase in pressure was not achieved even with increased adrenaline doses added to the infusion pump. The condition progressed with severe anuria and persistent metabolic acidosis. The baby rapidly deteriorated and died 72 hours after surgery.

## 3 Discussion

POC is an extremely rare syndrome with a poor prognosis.^[1]^ POC includes 5 congenital defects, namely, a midline epigastric abdominal wall defect, defects affecting the lower sternum, anterior diaphragm, diaphragmatic pericardium, and various intracardiac defects.^[3]^ The hallmark of POC is an omphalocele associated with EC. Toyama also described the probable (incomplete) syndrome variant that includes 4 defects.^[4]^ Our study represents the first reported case of one of a set of dizygotic twins with the complete POC in Bosnia and Herzegovina. The presence of this complex anomaly in twins is extremely unusual, but individual case reports have been published.^[7,8]^ The etiology of POC is still unknown and is considered to be of a heterogeneous origin. It is believed that the lack of fusion in the medial line of the mesoderm is responsible for the development of this complex anomaly.^[9]^ It usually occurs sporadically, but familial cases have been reported indicating the potential role of genetic factors in the pathogenesis of POC.^[10]^ Since no etiological factor has been identified in our case, we can only hypothesize that hereditary and environmental factors, individually or combined, could be responsible for the occurrence of this anomaly.

Based on the number of malformations present, POC is classified as complete or incomplete. Class I or certain diagnosis implies a complete syndrome with all 5 defects present; Class II is a probable diagnosis, with 4 defects present (including intracardiac and ventral abdominal wall abnormalities); and class III is an incomplete syndrome, with various combinations of defects present, but always including a sternal abnormality.^[3]^ Accordingly, our patient fits class I or complete POC because all 5 components were present.

POC abdominal wall defects can be presented as omphalocele, epigastric hernia, umbilical hernia, diastasis recti, and combined defects.^[11]^ The most common abdominal wall defect is omphalocele. Our patients had a giant cephalic (epigastric) omphalocele in which the liver was centrally and mostly extracorporeally located. Abnormalities affecting the sternum can range from the short sternum, bifid sternum, absent xiphoid, and defective lower part of the sternum to the sternum's complete absence. In our case, the sternum was absent with only a small sternal ossification center. Defects in the heart pericardium, mostly in the diaphragmatic pericardium, are integral components of the full spectrum of POC syndrome and the anterior diaphragmatic hernia, which in our case was bilateral. Failed differentiation of the mesoderm into the septum transversum during early fetal development is considered the reason for the emergence of POC components. Various heart defects have been reported in infants with POC, including VSD, ASD, pulmonary stenosis, dextrocardia, and tetralogy of Fallot. The most severe cardiac anomaly in POC patients is EC. Our patient also had EC with the heart located within the abdominal cavity, to the right of the medial line, below the omphalocele sac. The findings that VSD (100%) and ASD (53%) are the most common intracardiac anomalies in POC were also confirmed in our case.^[3]^

POC management represents a tremendous multidisciplinary challenge involving pediatric surgery, cardiovascular surgery, anesthesiology, and NICU.^[12]^ Early surgical correction is the preferred treatment modality to minimize the risk of heart complications and reduce fluid loss and infection onset due to uncorrected omphalocele.^[13]^ However, early surgical intervention might be associated with an increased mortality rate.^[13]^ The usefulness of initial conservative treatment in stable patients has also been shown by using antibiotic prophylaxis and dressing with topical antimicrobial agents of the omphalocele sac until its epithelialization. In the present case, based on the multidisciplinary approach, delayed surgery was planned and performed.

Postoperative intensive care is essential to prevent or reduce hypoxia-related complications caused by pulmonary hypoplasia, surgical corrections of congenital malformations, and increased intra-abdominal pressure after the closure of the anterior abdominal wall.

POC is considered a high-mortality syndrome. Survival is related to the severity and complexity of heart malformations and EC location.^[14]^ Late mortality is caused by complications of heart dysfunction, infections, or adhesive small bowel obstruction.^[14,15]^ Due to the patient's preoperative unstable status and the heart's postoperative inability to adjust to the intrathoracic conditions, our patient died shortly after surgery.

In conclusion, POC is a high-mortality syndrome with a poor prognosis, particularly in patients affected by the POC complete form. Timely identification of all aspects of this syndrome, supportive care, and the choice of an optimal surgery modality (primary/staged) are the unique challenges of a multidisciplinary team in managing patients with POC.

## Author contributions

**Conceptualization:** Zlatan Zvizdic, Semir Vranic.

**Data curation:** Zlatan Zvizdic, Irmina Sefic Pasic, Amira Mesic, Sabina Terzic, Semir Vranic.

**Formal analysis:** Zlatan Zvizdic, Irmina Sefic Pasic, Semir Vranic.

**Funding acquisition:** Semir Vranic.

**Investigation:** Zlatan Zvizdic, Irmina Sefic Pasic, Amira Mesic, Sabina Terzic.

**Writing – original draft:** Zlatan Zvizdic, Semir Vranic.

**Writing – review & editing:** Zlatan Zvizdic, Semir Vranic.
